# Audit of the Completion Rate of BPD Admission Checklist for the Hospital Admitted Service Users With EUPD

**DOI:** 10.1192/bjo.2024.540

**Published:** 2024-08-01

**Authors:** Ranjan Baruah, Simon Graham, Jehan Elturky, Hannah Ruth, Faraaz Abulais

**Affiliations:** 1 Mersey Care NHS Foundation Trust, Liverpool, United Kingdom; 2 Mersey Care NHS Foundation Trust, London, United Kingdom

## Abstract

**Aims:**

As admissions have the potential to contribute to iatrogenic harm, Mersey Care NHS Foundation Trust (MCFT) introduced an admission checklist to help the decision-making process around admitting people with Borderline Personality Disorder (BPD).

1. To conduct an audit to review if the admission checklist was being used after its introduction.
2. To provide data on the context of admission including the use of MHA.

**Methods:**

Data from admissions for people with BPD to nine acute care wards in (MCFT) over a three-month period were collected and assessed for 21 parameters.

A total of 60 admissions were identified for 51 patients (9 patients had more than one admission).

**Results:**

None of the recorded 60 admissions had a completed BPD checklist at the time of admission.

36 (60%) of the decisions to admit took place during the Normal Working Hours (NWH), 24 (40%) out of hours (OOH).

33 (55%) informal admissions, 27 (45%) on Section 2 of the MHA.

NWH admissions were associated with a higher number of informal admissions compared with OOH admissions (24 vs 9 respectively).

3 out of 27 OOH admissions requested by Crisis Resolution and Home Treatment (CRHT) resulted in informal admissions. The remaining OOH admissions were following a Mental Health Act Assessment (MHAA) by trainee psychiatrists.

At the point of admission, 9 (15%) patients were not open to secondary mental health team in MCFT prior to their referral for MHAA; 48 (80%) patients were under Community Mental Health Teams and/or the CRHT; 12 (20%) were open to the Personality Disorder (PD) hub, and 3 (5%) were open to other mental health teams including eating disorders team, Attention Deficit Hyperactivity Disorder (ADHD), Addiction Services and Criminal Justice & Liaison Team (CJLT).

**Conclusion:**

There was no engagement with completing the BPD admission checklist. 40% of ST doctors reported on a separate survey that they cannot locate the Checklist on patient information system.

Admission decisions made during NWH have led to significantly more informal admissions compared with during OOH where the MHA was more likely to be used.

An action plan was designed to improve engagement with the admission checklist:

- Introductory training was provided to CRHT, approved mental health professionals (AMHPs), MHA second opinion doctors and psychiatry ST doctors.
- Inpatient teams were asked to complete the checklist.
- Bed Management to request an updated completed PD admission Checklist prior to admission.
- Re-auditing in 6 months.

